# The Protective Effect of Naringenin on Airway Remodeling after *Mycoplasma Pneumoniae* Infection by Inhibiting Autophagy-Mediated Lung Inflammation and Fibrosis

**DOI:** 10.1155/2018/8753894

**Published:** 2018-04-04

**Authors:** Yan Lin, Dan Tan, Qianna Kan, Zhen Xiao, Zhiyan Jiang

**Affiliations:** Department of Pediatrics, Longhua Hospital, Shanghai University of Traditional Chinese Medicine, 725 South WanPing Road, Shanghai 200032, China

## Abstract

Our previous study has shown that Chinese medicine, Qingfei Tongluo formula (QTF), has a significantly therapeutic effect to *Mycoplasma pneumoniae* (MP) pneumonia (MPP). The aim of this study was to investigate the therapeutic effect and mechanism of naringenin (NRG) on MPP which was an important component of QTF. Here, we studied 124 children with or without MPP and compared inflammatory cytokines and fibrinogen-related protein expression with enzyme-linked immunosorbent assay. We also employed a BALB/c mouse model of MPP and divided the mice into three groups: ctrl (normal control mice), MPP (MP*-*infected mice), and MPP + NRG (MP*-*infected mice treated with NRG). BEAS-2B cells were used to confirm the relationship between autophagy, inflammation, and fibrosis. The results show proinflammatory cytokines (interleukin- [IL-] 6, IL-1*β*, and tumor necrosis factor-*α*), and transforming growth factor beta (TGF-*β*) expression was significantly increased after MP infection from both clinical and animal experiment. In vivo experimental confirmation showed that NRG treatment decreased MPP-induced lung injury in mice by inhibiting autophagy-mediated inflammatory cytokine expression and pulmonary fibrosis. In vitro experiments confirmed it. These results indicate that NRG treatment suppressed the inflammatory response and pulmonary fibrosis by inhibition of autophagy after MP infection.

## 1. Introduction

*Mycoplasma pneumoniae* (MP) is a common cause of community-acquired pneumonia, mainly in children and young adults, and is well known to cause a wide variety of respiratory and extrapulmonary diseases [1, 2]. Increasing evidence has confirmed that the symptoms of pneumonia caused by MP are correlated with the induction of proinflammatory cytokine expression and pulmonary fibrosis [3–5]. MP infection can lead to proinflammatory cytokine, tumor necrosis factor-*α* (TNF-*α*), and chemokines, such as interleukin- (IL-) 6, promoting the recruitment of various leukocytes, primarily neutrophils, to the site of infection, eventually leading to lung injury and pulmonary fibrosis.

Autophagy, a conserved eukaryotic stress-response pathway in which cells sequester damaged or surplus proteins and organelles in double-membrane vesicles and deliver them to lysosomes for degradation, also plays a seminal role in antimicrobial host defense. Classic triggers of autophagy include nutrient deprivation and inhibitors of the metabolic regulator mTOR, such as rapamycin [6]. Autophagy is also strongly induced by bacteria, viruses, and fungal organisms that ultimately are enclosed within autophagosomes and digested within the autophagolysosomes [7, 8]. Autophagy has been implicated in MP infection in cultured macrophages in vitro [3, 9]. But the relationship between MP-induced autophagy and inflammatory responses remains unknown.

Our previous study found that Chinese medicine, Qingfei Tongluo formula (QTF), has been used for the clinical treatment of *Mycoplasma pneumoniae* pneumonia (MPP) resulting in a significantly therapeutic effect. Increasing evidence has shown that naringenin (NRG), an important component of QTF, has a regulatory function to fibroblasts and the inflammatory response. Hernandez-Aquino et al. have found that NRG can prevent experimental liver fibrosis by blocking TGF-*β* signaling [10]. Hua et al. found that NRG pretreatment inhibits neuroapoptosis and ameliorates cognitive impairment in rats exposed to isoflurane anesthesia by regulating the PI3/Akt/PTEN signaling pathway and suppressing NF-*κ*B-mediated inflammation [11]. NRG also inhibits stress-induced autophagy [12] and has a protective effect on oxidative stress-induced lung damage [13]. This suggests that NRG can inhibit MP-induced inflammatory responses and pulmonary fibrosis by suppressed autophagy.

In this study, we hypothesized that NRG might reduce the inflammatory response, halt the progression of pulmonary fibrosis, and contribute to the improvement of lung function in a murine model of MPP by inhibiting autophagy. For this purpose, we have investigated the mechanisms of action of NRG and its potential efficacy in the treatment of MPP.

## 2. Materials and Methods

### 2.1. Clinical Specimen Collection and Ethics Statement

Peripheral blood samples were obtained from 60 patients with MPP and from 64 healthy volunteers (Table 1). A standard format elaborated by the principal investigator with clinical features was included for age, sex, and relative inflammatory factors and fibrinogen-related protein. Permission to use the samples for research purposes was obtained and approved by the Ethics Committee of the Longhua Hospital of China, and a written consent was obtained from all patients.

### 2.2. MP Culture

MP strain ATCC15531 (American Type Culture Collection, Rockville, MD, USA) was cultured in modified Hayflick medium (GZBIOTEST Co. Ltd., Guangdong, China) containing PPLO broth, horse serum, and 25% yeast extract, which was added along with penicillin G (1,000 U/ml), thallium acetate (0.025%), glucose (0.5%), and phenol red (0.002%) at pH 7.6. MP was cultured at 37°C in 5% CO_2_ for 7 days.

### 2.3. BEAS-2B Cell Culture and Viability

**Figure 1:**
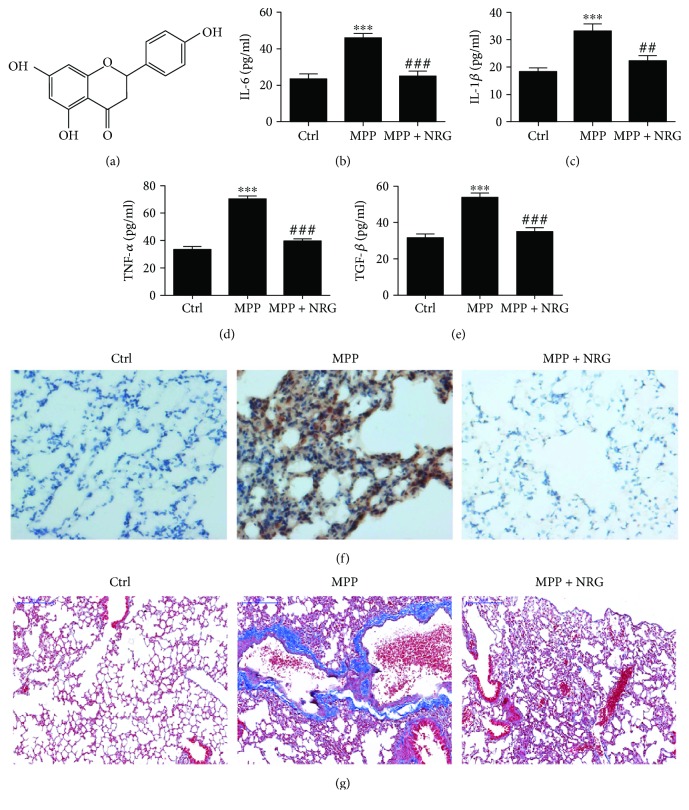
NRG treatment decreased *M. pneumonia*-induced lung injury in mice by inhibiting inflammatory cytokine expression and pulmonary fibrosis. (a) Chemical structure of NRG. (b–d) Inflammatory cytokines IL-6 (b), IL-1*β* (c), TNF-*α* (d), and fibrosis factor TGF-*β* (e) concentrations in mice serum were measured with ELISA. Data are presented as the mean ± SD with Tukey–Kramer multiple comparison test analysis. *n* = 5. ^∗∗∗^*P* < 0.001 versus ctrl group; ^##^*P* < 0.01, ^###^*P* < 0.001 versus MPP group. (f and g) Immunohistochemistry shows the presence of apoptosis (TUNEL staining) (f) and fibrosis (Masson trichrome staining) (g) in lung tissues. The results show that NRG treatment significantly suppressed the MPP-induced apoptosis and fibrosis. In Masson trichrome staining group, the collagen fibers were stained in blue. In TUNEL staining group, the nucleus of apoptosis cell was stained in brown.

The human lung epithelial BEAS-2B cell line (purchased from Procell Life Science Co. Ltd., Wuhan, China) which stably expressing GFP-LC3 [14] was cultured at 37°C in a humidified atmosphere with 5% CO_2_ in Dulbecco's modified Eagle's medium/F12 medium supplemented with 10% fetal bovine serum, 100 U/ml penicillin, and 100 ng/ml streptomycin (Invitrogen, Tokyo, Japan). To identify the effect of NRG on MP-induced airway fibrosis and inflammation, BEAS-2B cells were treated with NRG (100 *μ*M, [Figure 1(a)]) or 3-methyladenine (3-Ma, 4 mM) (purchased from Sigma-Aldrich [St. Louis, MO, USA]) for 2 h prior stimulated with 1 × 10^6^ cfu (colony-forming units) MP for up to 24 h. The control group was treated with normal saline. Then BEAS-2B cells were collected for further study.

### 2.4. Establishment of the MP Infection Model

Four-week-old BALB/c mice (mean body weight, 15 ± 1 g) were purchased from Shanghai Sippr Bk Laboratory Animals Co. Ltd. (Shanghai, China) and housed in a specific-pathogen-free environment with ad libitum access to food and water. After anesthesia, all mice were divided randomly into three groups: ctrl (normal control mice), MPP (MPP-infected mice), and MPP + NRG (MP-infected mice treated with NRG). On day 1, mice in the ctrl and MPP groups were treated with normal saline by intragastric administration, whereas mice in the MPP + NRG group were treated with NRG (100 mg kg^−1^) by intragastric administration. Mice in all groups were subjected to anesthesia with 0.5% pentobarbital sodium at a concentration of 0.25 ml/20 g. Then mice in the MPP and MPP + NRG groups were inoculated intranasally with 50 *μ*l of MP at ~1 × 10^8^ cfu as reported in a previous study [15]. A 50 *μ*l inoculation of saline was similarly given to the mice in the control groups. On day 2, the foregoing steps were repeated. On days 3 to 7, mice in the MPP + NRG group were treated with only NRG. Mice in the MPP group or ctrl group were treated with 0.25 ml of normal saline by intragastric administration repeated for three weeks.

After 1 month of treatment, the mice were intraperitoneally anesthetized with 0.05 ml of 10% chloral hydrate and fixed in the supine position before sample collection. The neck and chest were sterilized with conventional iodine and alcohol, and then the chest was opened with sterile equipment (soaked in 0.1% diethylpyrocarbonate for 24 h and then autoclaved). The entire lung was collected and stored in liquid nitrogen. Serum was isolated from peripheral blood. The animal study protocol was approved by the Ethics Committee of Longhua Hospital.

### 2.5. Transmission Electron Microscopy

To examine the formation of autophagosomes in lung tissue, transmission electron microscopy was performed as previously described [16]. Briefly, lung tissue (1 mm^3^) from each group was fixed with ice-cold glutaraldehyde (2.5% in 0.1 M cacodylate buffer, pH 7.4) for 1 h, postfixed in OsO_4_, and embedded in epoxy resin. Ultrathin sections (70–80 nm) were stained with uranyl acetate and lead citrate and examined in a CM-120 electron microscopy (Philips, Holland).

### 2.6. Enzyme-Linked Immunosorbent Assay

TGF-*β*, IL-6, IL-1*β*, and TNF-*α* in the supernatants of BEAS-2B or serum from subjects with MPP or healthy volunteers were measured using human ELISA kits (Sen-Xiong Company, Guangdong, China); TGF-*β*, IL-6, IL-1*β*, and TNF-*α* in serum from mice with different treatment were measured using commercially available mice ELISA kits (Sen-Xiong Company, Guangdong, China). In accordance with manufacturer's instructions, supernatants were stored at −80°C before measurement and both standards and samples were run in triplicate. The OD_450_ was calculated and standard curves were plotted.

### 2.7. Western Blotting Analysis

Western blotting was performed using lung tissue homogenates in urea buffer (8 M urea, 1 M thiourea, 0.5% CHAPS, 50 mM dithiothreitol, and 24 mM spermine). Protein fractions were prepared using a protein extraction kit (Pierce, Waltham, MA, USA) following manufacturer's protocols. GAPDH was used as a loading control. Samples (40 *μ*g total protein) were separated with SDS-PAGE and transferred to nitrocellulose membranes (Millipore, Billerica, MA, USA). After blocking in 5% nonfat milk for 1 h, membranes were incubated with primary antibodies against *α*-SMA, collagen I, collagen III, Beclin-1, LC-3, P62, TGF-*β*, and GAPDH at 4°C overnight. After washing, membranes were incubated with horseradish peroxidase-conjugated secondary antibodies for 1 h at room temperature. Signals were detected using an ECL detection system (GE Healthcare, Chicago, IL, USA) and analyzed using ImageJ 1.42q software (National Institute of Health, Bethesda, USA). The in vitro experiment was independent repeated three times. The in vivo test was repeated three times in each animal.

### 2.8. Measurement of Cell Viability via MTT Assay

For the determination of cell viability, BEAS-2B cells were treated with NRG (0, 25, 50, 100, and 250 *μ*M) for 24 h and then cultured with MTT solution (5 mg/ml) (Sigma, St. Louis, MO, USA) for 4 h. The viable cells converted MTT to formazan, which generated a blue purple color after dissolving in 150 *μ*l of dimethyl sulfoxide. The absorbance at 590 nm was measured using a Multiskan™ GO microplate spectrophotometer (Thermo Fisher Scientific, Inc., Cleveland, USA). All assays were repeated as independent experiments at least twice.

### 2.9. Flow Cytometric Determination of Cell Death

Cell death was analyzed using an annexin V apoptosis detection kit (eBioscience, California, USA) as previously described [17]. Briefly, both floating and adherent BEAS-2B cells were collected and resuspended in 100 *μ*l of annexin V-binding buffer. After staining with 5 *μ*l of FITC-conjugated annexin V for 15 min at room temperature in the dark, the cells were washed and incubated with propidium iodide (PI). All cell samples were assayed using a FACSAria flow cytometer (BD Biosciences, New Jersey, USA), and the acquired data were further analyzed using FCS Express (De Novo Software, California, USA).

### 2.10. Autophagy Assay

Autophagy was determined by detection of the processing of the autophagy marker LC3 and fluorescence microscopic detection of the formation of the autophagosomes in cells transfected with GFP-LC3.

### 2.11. Immunohistochemistry Assay

Lung tissue samples were fixed in 10% formalin solution and embedded in paraffin. 5 *μ*m sections were stained with TUNEL and Masson's trichrome. Sections were examined using an Axiophot light microscope (Zeiss, Oberkochen, Germany) and photographed with a digital camera.

### 2.12. Statistical Analysis

Results are expressed as the mean ± standard deviation (SD). Statistical significance was evaluated by analysis of variance followed by Tukey–Kramer multiple comparison test and by Student's *t*-test. *P* < 0.05 denotes statistical significance.

## 3. Results

### 3.1. Comparison of Clinical and Laboratory Characteristics between Children with and without MPP

The characteristics of the subjects are shown in Table 1. There was no difference with respect to the mean age and sex among the two groups. The results show that children with MP pneumonia have a higher ratio of neutrophils and white blood cells in whole blood cell analysis. There was no significant difference in lymphocytes and blood platelets between the two groups. This suggests that MP infection can induce the inflammatory response.

The serum cytokine levels (IL-6, IL-1*β*, and TNF-*α*) were significantly higher in children with MP pneumonia than in those with nonatopic MP pneumonia (*P* < 0.05). The serum TGF-*β* levels were also significantly higher in children with MP pneumonia, suggesting that MP infection can induce pulmonary fibrosis because TGF-*β* plays an important role in mediating pulmonary fibrosis [18, 19].

### 3.2. NRG Treatment Decreased *M. pneumonia*-Induced Lung Injury in Mice by Inhibiting Inflammatory Cytokine Expression and Pulmonary Fibrosis

As shown in Figure 1, in *M. pneumonia* challenged mice, serum IL-6 (Figure 1(b)), IL-1*β* (Figure 1(c)), and TNF-*α* (Figure 1(d)) were significantly higher in mice after 1 month infection than their saline controls. NRG treatment decreased MP-induced inflammatory cytokine expression after MP infection in mice serum. ELISA has shown that NRG treatment suppressed MP-induced TGF-*β* expression (Figure 1(e)) suggesting that NRG can inhibit MP-induced fibrosis as has been previously reported [20]. Immunohistochemistry has shown that NRG treatment suppressed MP-induced apoptosis (Figure 1(f)) and pulmonary fibrosis (Figure 1(g)).

### 3.3. NRG Treatment Decreased *M. pneumonia*-Induced Autophagy

**Figure 2:**
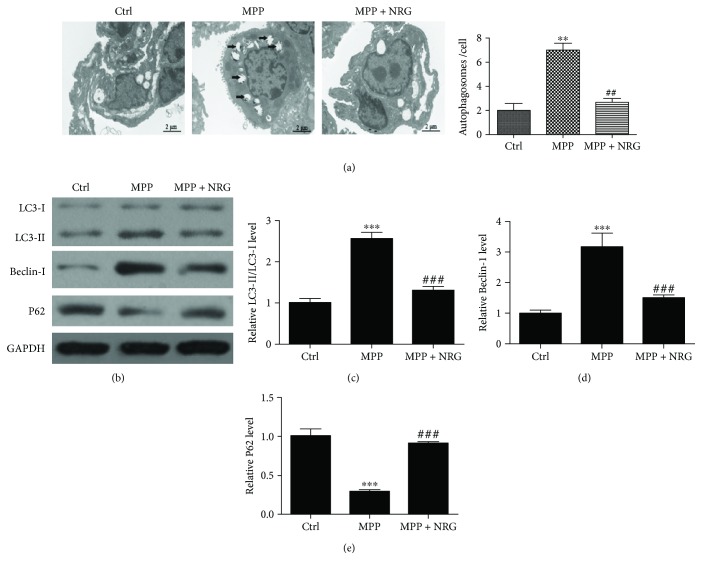
NRG treatment decreased *M. pneumonia*-induced autophagy. (a) TEM shows the autophagosome in lung tissues. Data are presented as the mean ± SD with Tukey–Kramer multiple comparison test analysis. *n* = 10. ^∗∗^*P* < 0.01 versus ctrl group; ^##^*P* < 0.01 versus MPP group. (b) Western blot detection shows the expression of autophagy relative protein LC3, P62, and Beclin-1. (c–e) The results are expressed as relative ratios of band density of LC3-II/LC3-I (c), Beclin-1 (d), and P62 (e). Data are presented as the mean ± SD with Tukey–Kramer multiple comparison test analysis. *n* = 3. ^∗∗∗^*P* < 0.001 versus ctrl group; ^###^*P* < 0.001 versus MPP group.

In order to identify whether MPP can induce autophagy and to verify the protective effect of NRG on MPP-induced lung inflammation and fibrosis relative to autophagy after MP infection and treatment with NRG for 1 month, lung tissues were collected for transmission electron microscope (TEM) analysis. The results of TEM show that the autophagosomes in lung tissues were increased after MP infection and that NRG treatment inversed the progress (Figure 2(a)). Western blot detection shows the expression of autophagy relative protein LC3, P62, and Beclin-1 (Figures 2(b)–2(e)). The results demonstrate that NRG suppressed MP-induced autophagy relative protein LC3 and Beclin-1 expression and increased MP-induced P62 inhibition. The conversion of LC3-I into LC3-II is an essential step in autophagosome formation, and the abundance of LC3-II correlates with the number of autophagosomes, suggesting a protective effect of NRG on MP-induced inflammation and fibrosis relative to autophagy regulation.

### 3.4. NRG Treatment Inhibits MP-Induced BEAS-2B Cells Injury by Inhibiting Autophagy

**Figure 3:**
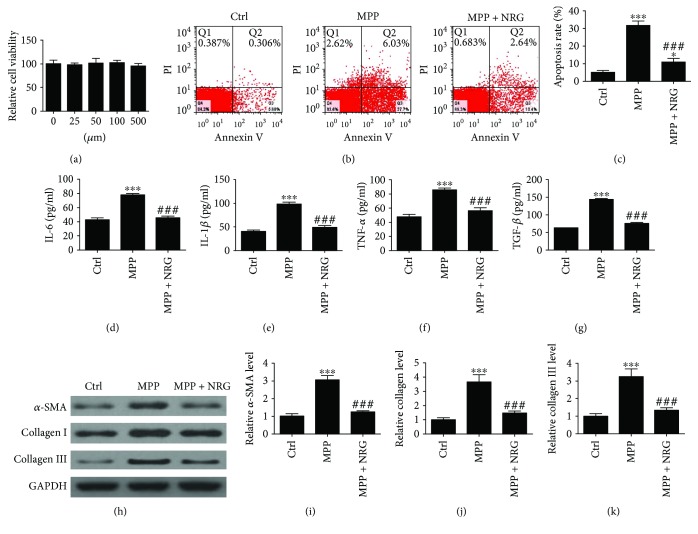
NRG treatment inhibits MP-induced BEAS-2B cells injury. (a) MTT analysis shows that NRG treatment has no significant effect on cell viability with different concentrations (*P* > 0.05). (b and c) Apoptosis of BEAS-2B cells was measured with FITC-annexin V/PI staining and flow cytometry in response to different treatments. Data are presented as the mean ± SD with Tukey–Kramer multiple comparison test analysis. *n* = 3. ^∗^*P* < 0.05, ^∗∗∗^*P* < 0.001 versus ctrl group; ^###^*P* < 0.001 versus MPP group. (d–f) Inflammatory cytokines IL-6, IL-1*β*, and TNF-*α* expression in cell supernatant were measured with ELISA. (g) TGF-*β* expression in cell supernatant was measured with ELISA. Data are presented as the mean ± SD with Tukey–Kramer multiple comparison test analysis. *n* = 5. ^∗∗∗^*P* < 0.001 versus ctrl group; ^###^*P* < 0.001 versus MPP group. (h–k) Fibrotic correlation factor *α*-SMA, collagen I, and collagen III expression in cell were measured with western blot. Data are presented as the mean ± SD with Tukey–Kramer multiple comparison test analysis. *n* = 3. ^∗∗∗^*P* < 0.001 versus ctrl group; ^###^*P* < 0.001 versus MPP group.

To confirm the relationship between autophagy, inflammation, and fibrosis, BEAS-2B cells were employed. MTT analysis shows that NRG treatment has no significantly effect on cell viability with different concentrations (0, 25, 50, 100, and 500 *μ*M), suggesting that NRG has no cytotoxicity (Figure 3(a)). FITC-annexin V/PI staining and flow cytometry analysis show that the apoptosis ratio of BEAS-2B cells was increased after MP infection for 24 h. NRG treatment (100 *μ*M) reversed MP-induced BEAS-2B cells apoptosis (Figures 3(b) and 3(c)). Inflammatory cytokines IL-6, IL-1*β*, TNF-*α*, and transforming growth factor beta (TGF-*β*) expression in cell supernatant were measured with ELISA (Figures 3(d)–3(g)). The results show that NRG can significantly inhibit MP-induced inflammatory cytokines and fibrin-related protein TGF-*β* expression. The results are consistent with previous clinical and animal experiments. Western blot confirmed that fibrotic correlation factor *α*-SMA, collagen I, and collagen III expression in BEAS-2B cells were decreased with NRG treatment.

**Figure 4:**
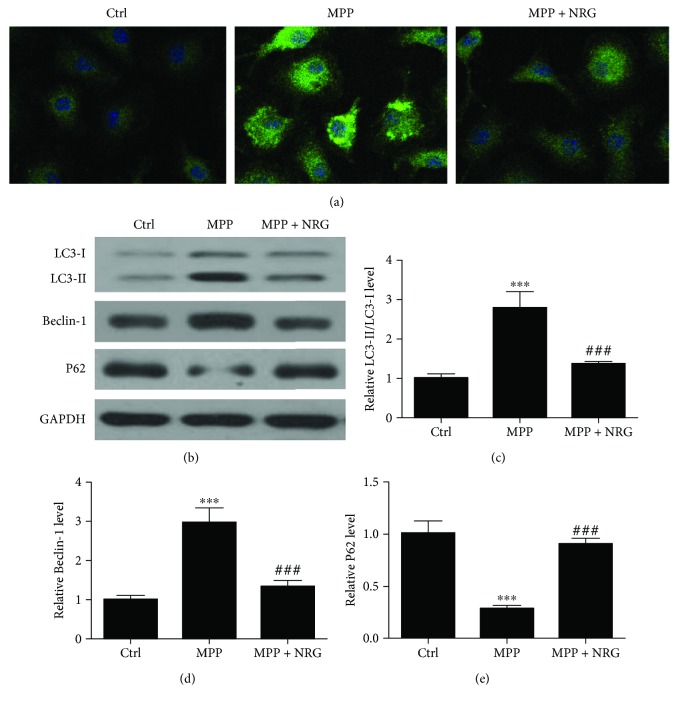
NRG treatment inhibits *M. pneumonia*-induced autophagy. (a) Immunofluorescence shows the effect of NRG treatment on MP-induced autophagy plaque production. (b–e) Western blot detection shows the expression of autophagy relative protein LC3, P62, and Beclin-1 expression. Data are presented as the mean ± SD with Tukey–Kramer multiple comparison test analysis. *n* = 3. ^∗∗∗^*P* < 0.001 versus ctrl group; ^###^*P* < 0.001 versus MPP group.

To identify the effect of NRG on MP-induced autophagy, BEAS-2B cells were transfected with LC3-GFP overexpression vector prior to MP infection (20 ng/ml) and NRG treatment. Immunofluorescence shows that NRG treatment reversed MP-induced autophagy plaque production (Figure 4(a)). Western blot detection confirmed that NRG inhibits MP-induced autophagy relative protein LC3 and Beclin-1 expression, but increased P62 expression (Figures 4(b)–4(e)).

### 3.5. Autophagy Inhibitor 3-Ma Treatment Inhibits *M. pneumonia*-Induced BEAS-2B Cell Injury

**Figure 5:**
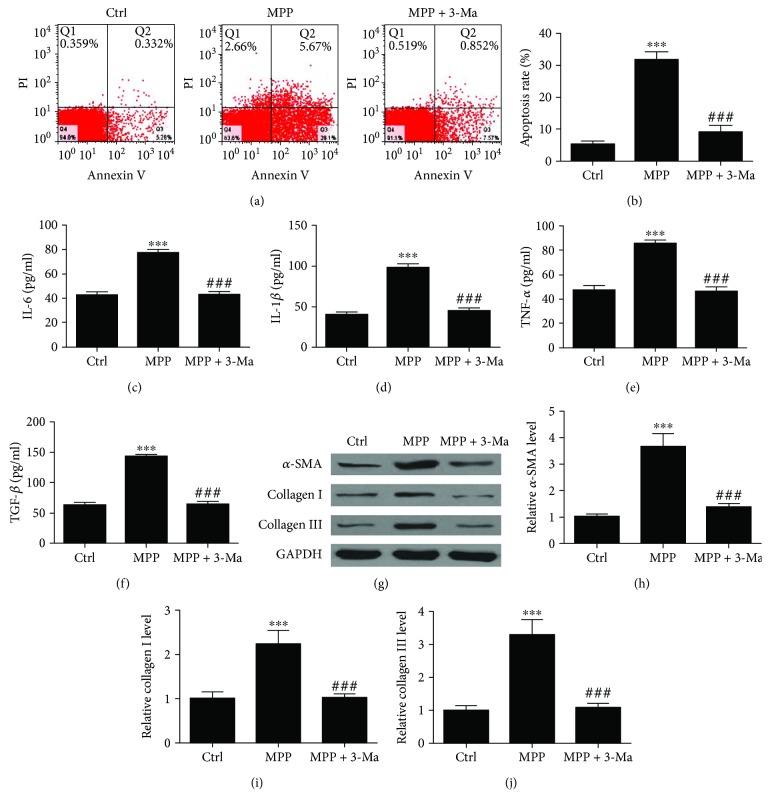
Autophagy inhibitor 3-Ma treatment inhibits *M. pneumonia*-induced BEAS-2B cells injury. BEAS-2B cells treated with 3-Ma (4 mM) for 2 h prior stimulated with 20 ng/ml MP for up to 24 h. (a and b) Apoptosis of BEAS-2B cells was measured with FITC-annexin V/PI staining and flow cytometry. Data are presented as the mean ± SD with Tukey–Kramer multiple comparison test analysis. *n* = 3. ^∗∗∗^*P* < 0.001 versus ctrl group; ^###^*P* < 0.001 versus MPP group. (c–e) Inflammatory cytokines IL-6, IL-1*β*, and TNF-*α* expression in cell supernatant were measured by ELISA. Data are presented as the mean ± SD with Tukey–Kramer multiple comparison test analysis. *n* = 5. ^∗∗∗^*P* < 0.001 versus ctrl group; ^###^*P* < 0.001 versus MPP group. (f) TGF-*β* expression in cell supernatant was measured with ELISA. Data are presented as the mean ± SD with Tukey–Kramer multiple comparison test analysis. *n* = 5. ^∗∗∗^*P* < 0.001 versus ctrl group; ^###^*P* < 0.001 versus MPP group. (g–j) Fibrotic correlation factor *α*-SMA, collagen I, and collagen III expression in cell were measured with western blot. *n* = 3. ^∗∗∗^*P* < 0.001 versus ctrl group; ^###^*P* < 0.001 versus MPP group.

To further confirm the relationship between autophagy, inflammation, and fibrosis, BEAS-2B cells treated with 3-Ma (4 mM) for 2 h prior to treatment with 20 ng/ml MP for up to 24 h. FITC-annexin V/PI staining and flow cytometry analysis show that MP-induced apoptosis of BEAS-2B cells was reduced with autophagy inhibition (Figures 5(a) and 5(b)). Inflammatory cytokines IL-6, IL-1*β*, and TNF-*α* expression in cell supernatant were measured by ELISA. The results show that autophagy inhibition reversed MP-induced inflammatory cytokine expression (Figures 5(c)–5(e)). ELISA and western blot analysis also show that autophagy inhibition reversed MP-induced fibrosis-related protein expression (Figures 5(f)–5(j)). In conclusion, we found NRG inhibits MP-induced inflammation and pulmonary fibrosis by inhibiting autophagy.

## 4. Discussion

*Mycoplasma pneumoniae* causes primary atypical pneumonia, tracheobronchitis, pharyngitis, and asthma in humans [21, 22]. Moreover, 3%–64% of pediatric asthma patients in the chronic stable phase are infected with *M. pneumoniae*. Thus, MP is a known aggravating factor of asthmatic symptoms in both acute exacerbations and the chronic stable phase. This is further demonstrated by the alleviation of respiratory system symptoms and improvement of pulmonary function after use of macrolides to treat MP infection [23]. The symptoms of pneumonia caused by *M. pneumoniae* are correlated with the induction of proinflammatory cytokines [24] and induced pulmonary fibrosis [25], suggesting that the excessive immune responses induced by *M. pneumoniae* play an important role in the development of pneumonia.

The involvement of autophagy in the pathogenesis of asthma was demonstrated previously by microscopic examination showing autophagosome formation in fibroblasts from asthmatic patients [26]. Furthermore, increased airway responsiveness and inflammatory cytokine expression were associated with LC3 upregulation and increased autophagosome formation in eosinophils in an ovalbumin-induced asthma mouse model, suggesting the involvement of autophagy in allergic inflammation [27]. Increasing evidence shows that inhibiting autophagosome formation in airways can significantly suppress bronchial fibrosis and the inflammatory response [28, 29]. So, the modulation of autophagy may have the potential to treat common respiratory ailments and disorders.

Our previous study has shown that the Chinese medicine, QTF, has been used for the clinical treatment of MPP and with a significantly therapeutic effect. NRG, an important component of QTF, has been reported to play an important in anti-inflammatory and antifibrotic role [10, 30]. This study aimed to clarify the effect of NRG on MP-induced inflammatory and lung fibrosis in pneumonia. To investigate the relationship between autophagy, inflammation, and fibrosis, our study found that inflammatory and fibrosis-related protein expression was significantly increased in both clinical, in vitro, and in vivo experiments after MP infection. The results of in vitro and in vivo experiments also show that MP infection promotes autophagy. NRG treatment reversed MP-induced lung inflammation and fibrosis by inhibiting autophagy. The results were confirmed by 3-Ma treatment (an autophagy inhibitor). Based on the data presented, we have shown the protective effect of NRG on airway remodeling after MP infection by inhibiting autophagy-mediated lung inflammation and fibrosis.

## Figures and Tables

**Table 1:** Characteristics of subjects in the study.

Demographic characteristics	Non-*Mycoplasma pneumoniae* (*n* = 64) (%)	*Mycoplasma pneumoniae* (*n* = 60) (%)
*Sex*		
Female	34 (53%)	38 (63%)
Male	30 (47%)	22 (37%)
*Age*		
1–3 years	5 (7.8%)	4 (6.7%)
3–7 years	41 (64.1%)	32 (53.3%)
7–14 years	18 (28.1%)	24 (40%)
Average age	6.51 ± 2.85	7.01 ± 2.80
*Whole blood cell analysis*		
White blood cell (×10^9^)	10.82 ± 2.38	11.23 ± 3.24^∗^
Neutrophils (%)	28.79 ± 13.24	37.38 ± 12.68^∗∗^
Lymphocytes (%)	61.28 ± 14.26	57.36 ± 16.38
Blood platelet (×10^9^)	375.9 ± 132.7	399.8 ± 142.8
*Expression of mammary immune-associated factors*		
IL-1*β* (pg/ml)	23.67 ± 6.35	67 ± 11.33^∗∗∗^
IL-6 (pg/ml)	46.58 ± 8.67	95 ± 16.23^∗∗∗^
TNF-*α* (pg/ml)	56.34 ± 8.33	128.98 ± 14.27^∗∗∗^
TGF-*β* (pg/ml)	16.87 ± 5.34	32.45 ± 8.62^∗∗∗^

IL-17: interleukin- (IL-) 17; IL-1*β*: interleukin- (IL-) 1*β*; IL-6: interleukin- (IL-) 6; TNF-*α*: tumor necrosis factor-*α*; TGF-*β*: transforming growth factor-*β*. Data are expressed as mean ± SD with Student's *t*-test analysis. ^∗^*P* < 0.05, ^∗∗^*P* < 0.01, ^∗∗∗^*P* < 0.001 against the control values.
